# Out of sight, but not out of mind: a case study of the collaborative development of a university-wide orientation resource for online students

**DOI:** 10.1007/s11423-022-10090-3

**Published:** 2022-02-23

**Authors:** Filia Joanne Garivaldis, Jennifer Chung, Leah Braganza, Lilani Arulkadacham, Richa Sharma, Andrea Reupert, Stephen McKenzie, Geoffrey Rose, Timsy Gupta, Zahra Aziz, Tony Mowbray, Dragan Ilic, Matthew Mundy

**Affiliations:** 1 Monash Sustainable Development Institute, Monash University, 8 Scenic Boulevard, Clayton Campus, Melbourne, VIC 3800 Australia; 2 School of Psychological Sciences, Monash University, 18 Innovation Walk, Clayton Campus, Melbourne, VIC 3800 Australia; 3 Faculty of Education, Monash University, 19 Ancora Imparo Way, Clayton Campus, Melbourne, VIC 3800 Australia; 4 Department of Civil Engineering, Institute of Transport Studies, Monash University, 23 College Walk, Clayton Campus, Melbourne, VIC 3800 Australia; 5 School of Allied Health, Human Services & Sport, La Trobe University, Bundoora, Melbourne, VIC 3086 Australia; 6 School of Public Health and Preventative Medicine, Monash University, 553 St Kilda Road, Melbourne, VIC 3004 Australia; 7 Present Address: The University of Melbourne, Redmond Barry Building, Parkville, Melbourne, VIC 3010 Australia

**Keywords:** Online education, Orientation resource, Educational co-design, Community of practice, Online study support

## Abstract

The global online education sector has been rising rapidly, particularly during and after the events of 2020, and is becoming mainstream much sooner than expected. Despite this, research studies report higher levels of perceived isolation, difficulties with engagement, and higher attrition rates in online compared to equivalent on-campus programs. Reasons include restrictions to the type of institutional support accessible by online students, and the lack of comprehensiveness of orientation resources. This paper describes the collaborative efforts by a cross-faculty academic team, supported by a community of practice, to create a university-wide online orientation resource—the Monash Online Learning Hub (MOLH). The development of the MOLH involved multiple phases, including an analysis of current practice, resource design and content creation, formative evaluation by staff and students, and successful integration into the university’s mainstream student orientation platform for widescale implementation. The methods adopted were varied, and involved generating both qualitative and quantitative data across multiple phases of development from online education experts at the University, that culminated in the gradual building and refinement of the MOLH. Final outcomes, implications and lessons learned are also discussed in this paper.

## Introduction

“Imagine… that you go to a University where all of the buildings are empty—no desks, tables, or chairs, just big bulletin boards all over each room” (Hamilton & Zimmerman, 2002, cited in Holley & Oliver, 2010). With this image, Hamilton and Zimmerman (2002) offer an alternative view of university life—a view that is said to be owned by students studying in a virtual space. Fortunately, rapid advances in educational technology since the early 2000s have improved online teaching and learning far beyond this rather dystopian view (Palvia et al., 2018). This now sought-after mode of teaching offers a rich, diverse and flexible teaching experience suited to many learners who would otherwise be unable to access higher education due to distance from a physical campus or time constraints (Norton & Cakitaki, 2016; Norton & Cherastidtham, 2014).

There remain, however, some natural and significant differences in the experiences of online versus on-campus students. Not least, a lack of physical infrastructure, or physical presence of a university campus, can fuel the need for some other form of connection to peers and university services (Roddy et al., 2017). More specifically, a review of the growing online education literature has revealed the most common, albeit non-exclusive, online student needs, displayed in Table 1. Identifying these needs is critical as the online mode of delivery of education becomes mainstream.

**Table 1:** Summary of online student needs based on a review of existing literature

Need	Description	Source
a	Technical competence to learn and study online and using university dashboards and LMS	Cho (2012), Horvath et al. (2019), Taylor et al. (2015)
b	Confidence in one’s own skills to use the LMS to learn and interact with course materials, and communicate with others	Cho (2012), Horvath et al. (2019)
c	Organisational skills and effective time/task management of online study	Bolliger and Martindale (2004), Broadbent and Poon (2015), Greenland and Moore (2014), Horvath et al. (2019), Johnson (2015), Khiat (2015)
d	Building resilience	Johnson (2015), Khiat (2015)
e	Motivation to study and academic self-efficacy	Lee and Choi (2011) as cited in Cho (2012)
f	Perceived isolation, lack of engagement with peers and teachers, and feelings of sense of community	Horvath et al. (2019), Roddy et al. (2017)
g	Academic writing, referencing and assignment preparation	Mupinga et al. (2006)
h	Career and employability	Eaton et al. (2018), Horvath et al. (2019)
i	Student mental health and support resources	Eaton et al. (2018), Roddy et al. (2017)

The needs listed in Table 1 either relate to university services that would otherwise be offered to students on campus, such as career and employability services, or concern unique study needs that become particularly prominent in the online mode, such as resilience and self-efficacy.

One way in which institutions have sought to address these needs, to create and maintain a sense of presence and connection with their students, alongside an introduction to the services on offer, is through student orientation programs for new learners. Orientation programs, irrespective of study mode, welcome students and are proactive in attending to students’ needs, which can differ and sometimes be more pronounced, than the needs of students studying on-campus (Roddy et al., 2017). Carefully designed orientation programs delivered in the early weeks of online students’ enrolment have proven to reduce the attrition rates that are commonly high in this study mode (Gazza & Hunker, 2014; Jones, 2013; Mitchel, 2014; Rovai & Wighting, 2005; Simpson, 2004; Tomei et al., 2009). Risk factors associated with attrition include a lack of experience studying online, a lack of organisational skills such as time management (Gaskell, 2006), personal attributes such as the inability to stay motivated, and inaccurate perceptions of the demands and requirements of study (Lee & Choi, 2011; Nash, 2005). As such, orientation programs may serve as an early intervention to help establish students’ realistic and accurate expectations of study, and may offer a means for prevention of problems escalating.

Beyond facilitating student retention, orientation programs have the potential to bring together, either physically or virtually, students from geographically dispersed locations, to establish a learning community and ease their transitions into university or college (Cannady, 2015). Orientation programs also enable students to become familiar with their learning environment, and for online students, their *online* learning environment (Cho, 2012), including its technical requirements. Indeed, technical difficulties are one of the biggest challenges online students face, particularly for those students who are of a mature age, and/or are returning to study after a long break (Roddy et al., 2017). With technical competence and confidence students can focus on their learning goals (Roddy et al., 2017). Finally, orientation programs can increase students’ general confidence and sense of belonging to the institution (Tomei et al., 2009).

As yet, the majority of orientation programs are offered to on-campus student cohorts (Cannady, 2015). Orientation programs for online students, instead, are fewer, and usually more piecemeal, split between course/degree-specific introductions, centred on departmental and faculty information, as well as library content. However, relying on individual efforts within an institution to deliver online orientation programs for online students could lead to these programs being narrow in their offerings, creating inequalities in student experiences across the same institution, and at times duplicating individual staff efforts to meet common individual student needs (as was found to be the case in this case study).

### Developing orientation resources for online students

As online courses grow in number and in size, institutions and their academics have recognised the significance of the issues discussed above, and have begun developing or consolidating orientation resources. For example, Cho (2012) describes the process adopted to develop an online student orientation (OSO) resource, targeted to supporting students planning to enrol in an online course, or who had already enrolled in an online course, in an American university. The site was intended to meet the needs of thousands of students (identified to be 3200 students in 2009). The development of the OSO involved several phases, beginning with the analysis phase. This phase involved a needs assessment to identify the goals of the OSO, a task analysis, to identify what online students needed to learn based on course syllabi and the online literature, as well as a context analysis, to decipher the generic format of existing online courses such that the new OSO could be created to simulate these courses. Following analysis, the design phase involved mapping out four modules of learning material to meet the resource’s objectives, which included developing an understanding of online learning, skilfully using the LMS for learning, independently solving technical issues encountered, and developing self-awareness about online learning skills. In particular, one of the modules comprised a self-assessment measure of student readiness, to help students determine their self-efficacy in successfully completing an online course. All content was validated via expert reviews from faculty members, and improvements were made based on feedback. The resource underwent both formative and summative evaluation, with both faculty members and students. Students indicated that they were overall satisfied with the OSO, and particularly its ease of navigation, its content and its accessibility, organisation and design. The most common theme that emerged from participants’ positive qualitative comments of the resource concerned its content around enhancing understanding of what it means to study online.

A different scope was adopted by Taylor et al. (2015) whereby a team of instructional designers, educators, and a program lead developed non-course-specific interactive videos with voice-over as a way of addressing the most common frustrations with technology of online students. The videos covered issues with LMS navigation, posting on discussion forums, submitting to a drop-box, and viewing and responding to assessment feedback. The videos were made available to students of courses with high attrition rates via their orientation programs. The authors report that the videos were viewed extremely positively by students, so much so that reductions in course withdrawal, and improvements in course grades, were observed.

More recently, Horvath et al. (2019) from a local university in Australia developed an orientation program across two phases, aiming to increase student preparedness for online study, feelings of belonging and a sense of community, as well as to facilitate ongoing support—topics which are often overlooked in online education (Horvath et al., 2019; Roddy et al., 2017). The development process for the program initially involved a review of current student barriers and drivers to online learning success (also discussed later in this paper). The second phase of the project involved the collaboration between academic staff from multiple disciplines, educational designers and library and other support staff, as well as information technology experts, in the design and development of the program. The team adopted a community of inquiry approach to create three orientation modules—Plan, Prepare, and Connect. The Plan module included welcome and orientation information, including a recorded message from the Vice Chancellor of the university. The Prepare module included information on the learning management system that students would be learning within, and university procedures and services including the student charter. The final module, the Connect module, included scheduled synchronous orientation sessions that featured teaching and support staff and student peer leaders, to establish connections and provide institution presence. Upon completion of the program, eight best-practice strategies were recommended for online orientation and transition programs, with student satisfaction, retention, and success in mind.

**Fig. 1:**
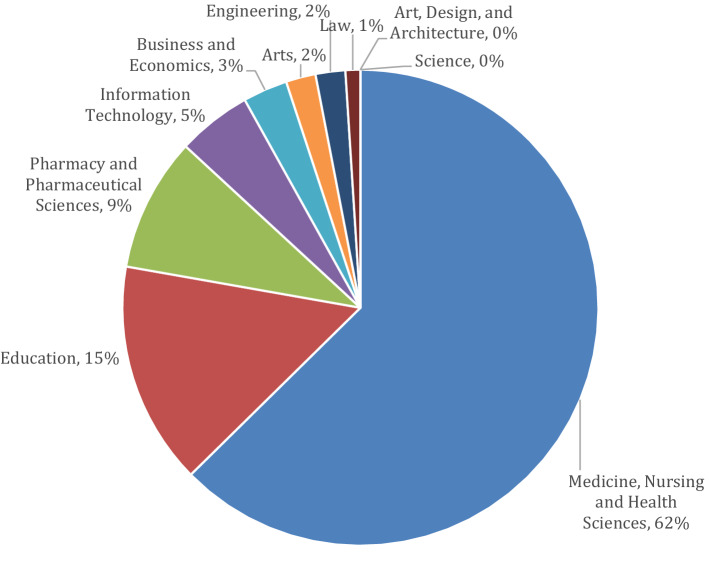
Proportion of students externally enrolled at Monash University by Faculty in 2018

Similarly, online education at Monash University has grown rapidly. According to a report generated by the Office of the Deputy Vice-Chancellor, 13,047 of the approximate 78,000 students enrolled at the university in 2018, studied in some capacity in the external online mode. The report also identified the discipline-specific profiles of the students, summarised in the Fig. 1.

The greatest proportion of online students at Monash University in 2018 were enrolled within the Faculty of Medicine, Nursing, and Health Sciences (*n* = 3724). These students come from both undergraduate and postgraduate courses of study, and a variety of study modes, i.e. ranging from the fully online intense mode, to a flexible blended mode. Based on this data, the university has seen an average annual growth in external enrolments of 1.5% and multi-modal enrolments of 9.8% (a total average of 5.5% annually) between 2014 and 2018.

As such, the need for an orientation resource purposely built for Monash University students is greater than ever. Helping to achieve this is a Community of Practice (CoP) that was formed and launched in 2018 comprising academic and support staff interested in online education from across the university, and which was specifically formed to address the needs of the growing body of online students. A working group of individuals from the community of practice and representing various faculties of the university, including the library, convened to develop the university’s first orientation site–the Monash Online Learning Hub (MOLH). Like the aforementioned orientation modules from other Universities, the MOLH aims to address students’ needs around online learning preparation, digital literacy, and academic and pastoral support through a community of practice development process. Unlike before, however, it was aspired that the MOLH would be discipline agnostic, applicable to all students studying online across the university, as well as address student well-being, a previously untouched aspect of the online student experience. This paper reports on the collaborative efforts implemented to develop MOLH, the processes followed to determine its content and interface, and the methods by which the resource was formatively evaluated. The paper ends with a discussion of lessons learned, and recommendations for a pilot study; a summative evaluation of the effectiveness of the MOLH resource after its initial implementation. We anticipate that the paper will present a valuable case study, offering other institutions and course developers another perspective on how to collaboratively produce their own online student resources.

## Case study: Developing the Monash Online Learning Hub (MOLH)

### Background and overview

Building the Monash Online Learning Hub (MOLH) was an initiative that involved a group of academic staff at Monash University who were members of the Monash Online Education Community of Practice (MOEC). Members of the CoP not only shared a common interest in collaboratively addressing the needs of the university’s growing community of online learners, but comprise individuals with extensive experience in the design, development, and delivery of online education, which is highly desirable in online resource development (Rose et al., 2020; Williams van Rooij & Zirkle, 2016). Members included senior members of the University, such as the Senior Pro-Vice Chancellor (Academic), academic faculty such as online educators and course leaders/coordinators, educational and learning designers, online teaching and learning innovation specialists, and library staff. Like other similar initiatives (Cho, 2012; Horvath et al., 2019; Taylor et al., 2015), the collaborative nature of the development of the MOLH provided an opportunity to create a more cohesive, integrated, complete and connected product, by a diverse team who is 'in touch' with the students and their needs that the resource is addressing. Access to the CoP, however, meant that the collaboration expanded beyond the project working group from the outset, and met further University needs in the area of engagement and community building (Rose et al., 2020). As such, the involvement of the CoP in this initiative enabled value creation—the opportunity to create a tangible and valuable outcome from the investments made by the CoP to support their existence (Wenger et al., 2000).

As such, the project was funded by a Monash University Inter-Faculty Transformation Grant, awarded by the Monash Education Academy in late 2018. The Faculties/Divisions of the university represented in the project working group, and the roles of these representatives included the course convenors and assistance course convenors of online courses from the Faculty of Medicine, Nursing, and Health Sciences, the Faculty of Engineering, the Faculty of Information Technology, and the Faculty of Education, a senior online learning advisor from the Library, an educational designer appointed later in the project, research assistants, and an online course graduate. The funded project involved the creation of the University’s first orientation site for all students studying in the online, blended, off-campus, distance and flexible modes, i.e. completing some part or all of their studies remotely. The project meets the institution’s Digital Education Direction Statement 2018–2022, which aims to provide “the opportunity to enhance exceptional learning experience, provide flexibility and nimbleness, as well as attract more students from non-traditional backgrounds”, in support of enhancing all modes of education. The site also takes guidance from the Australian Tertiary Education Quality Standards Agency (TEQSA), an accreditation body, which requires that students have “equivalent opportunities for successful transition into and progression through their course of study, irrespective of their educational background, entry pathway, or mode or place of study” (TEQSA, 2019, p. 1).

The site was envisioned to consolidate existing resources at the university, as well as create new resources, to provide efficient and sustainable solutions in the on-boarding or induction of online students. The site was to replace the duplication of individual/faculty efforts in developing resources with a dynamic tool assembled by a community of front-line staff, invested in the development and delivery of online education. Firstly, the site aims to meet the induction needs of new students, and provide ongoing study support material to continuing students, who may have limited or no access to a physical campus. Secondly, the site aims to foster and support a sense of community and belonging of students, and thus increase student engagement with their course and university.

In terms of the gains for the university, the site is expected to:

- Facilitate the sharing of online education relevant experience and knowledge between faculties, and foster collaboration with the aim of bringing best online practice and evidence-based resources for the direct benefit of students,
- Provide a centralised repository of online learning information and tools for students wanting to embark on online learning, such as online study readiness tools, allowing for a smooth and efficient transition from non-online learning modes, with associated space and cost savings, and most importantly,
- Ensure equity and inclusivity is achieved in the provision of resources, extended to all students, including the most remotely located.

### Method

**Fig. 2:**
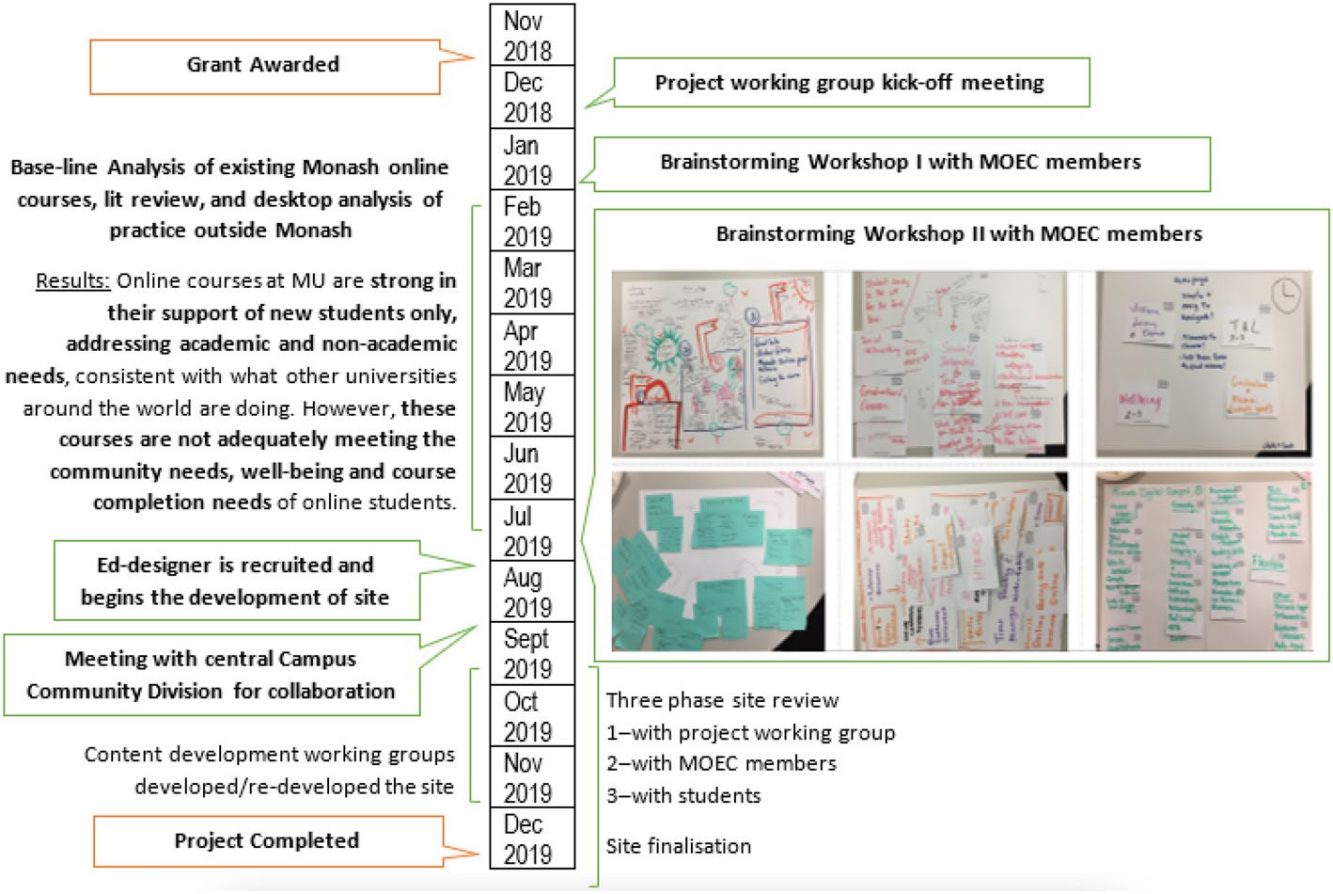
Project timeline and summary of main phases of development

As depicted in the Fig. 2, this project spanned over one year, starting from the end of the Australian academic year in December 2018 and ending in December 2019. An instructional systems design model (ISD) was applied across the several phases of the project, albeit in a non-linear fashion, including analysis, design, development, and evaluation steps, as used previously (Cho, 2012). Our process also aligns with a design-based research (DBR) approach, which advocates for the collaboration between researchers and practitioners in real-world settings, to create contextually-sensitive outcomes and resources (Reimann, 2010). The following figure outlines the steps we followed, and illustrates the flexible application of ISD and DBR.

The methods used to collect data follow the ISD model mentioned above, across four phases; (1) an analysis of baseline data in the early months of the project to understand current practice, (2) the design of the MOLH using insights and ideas generated by experts, (3) the development of the MOLH, and (4) an evaluation of an initial draft of MOLH by staff and students. Each of these phases of the methods are discussed separately in the following sections, as each phase’s outcomes feed into the phases that follow.

#### Analysis of baseline data to understand current practice

##### Materials

To create a baseline survey to be used to understand the current teaching and learning practices and resources implemented to support online education students at the university, a two hour workshop, “Brainstorming Workshop I”, was conducted with the CoP. In addition to this primary purpose, the workshop also served the CoP’s collaborative agenda, to establish relationships that would support the project in subsequent phases where input was to be critical, to improve the quality and standard of the resources developed, and to garner longer term support of the site starting from its launch and implementation (Wenger et al., 2000).

In total, 11 CoP members attended the workshop and shared their ideas and thoughts around the key resources that online students need to both facilitate their induction to the university, and to provide ongoing support throughout their online studies. The responses from this workshop could be grouped into discrete categories, with examples (see Table 2).

**Table 2:** Online learning resource themes and ideas generated for online students during the first CoP workshop

Category	Themes of resources	Examples
Course-specific resources	Assessment	Marking rubrics, academic integrity policy, submission policies
	Course overview content	Course structure and introductions to the teaching team, announcements and noticeboards
	Course specific refresher content	Prerequisite knowledge, instructional videos
	Course content structure in LMS	Moodle navigation, study guidelines
	Career progression	Industry knowledge, professional registration requirements, further study
General resources	Welcome	Keynote address, icebreakers, social networking platforms
	Study resources	Successfully learning online, overcoming challenges such as isolation, well-being and self-care resources including mindfulness
	Academic resources	Library resources, writing, critical thinking, etc.
	University resources	Student Charter, OHS guidelines, links to indigenous units
	Digital literacy	Moodle navigation, software use instructions, technology support services
	Careers	Graduation information and alumni

It was also suggested by participants in this workshop that care was needed with regard to the language used in the orientation site, the format and user-friendliness of the material, the ongoing accessibility of the site, and the sense of acknowledgement and belonging needs of the students. The outcomes of this workshop informed the baseline survey used in the second part of the analysis phase, which involved reviewing existing orientation resources offered at the university, discussed below.

##### Participants and procedure

This analysis aimed to gather insight into current online education practice within the university. The baseline analysis was conducted to identify orientation resources that already existed at Monash University, in recognition of existing areas of good practice, and to identify areas of unnecessary overlap and duplication of material.

A total of 40 online course convenors or educational designers, referred to as stakeholders, and who constructed, designed, and/or delivered online orientation resources to students of their respective online courses, were identified through the University’s Associate Deans of Teaching and Learning or the online course handbook, and were approached to take part in the baseline analysis.

Of the 40 stakeholders that were contacted in the middle of semester 1 in 2019 (April–May), 14 completed the survey (30% response rate). Of these 14, three reported that their courses did not offer any orientation resources to their students, whereas the remaining 11 participants reported offering some form of orientation to their online students. The results from these 11 participants are discussed here.

The majority of respondents that offer online orientation resources came from the Faculty of Medicine, Nursing and Health Sciences (*n* = 7), which also enrols the majority of online students, and one response was received from each of the Faculties of Engineering, Information Technology, Business and Economics, and Pharmacy. The orientation resources are mostly delivered to students via their learning management system (Moodle; *n* = 9), or via a website (*n* = 2).

Participants indicated with a “Yes” or “No” whether they provided each of the online learning resources listed in the baseline survey, summarised in Table 2., to their students. They also indicated how they rated the usefulness of each resource, irrespective of whether they offered it or not, on a 3 point Likert scale, where 1 = “Useful”, 0 = “Not sure”, and − 1 = “Not useful”. The most to least common orientation resources offered to students are displayed in Table 3.

**Table 3:** Orientation resources offered to online students, and the number of courses offering these resources, listed in order of most to least frequent

Resources	No. of courses offered	Resources (cont.)	No. of courses offered (cont.)
Navigating course material in LMS	8	Monash Student Charter	3
How to learn online	8	Online course handbook	3
General study instructions	8	Professional etiquette	3
Writing/Presentation skills	7	“Respect” module at Monash	3
Study planning	7	English language support services	3
Library resources	7	OH&S resources	3
Academic integrity policies	7	Making connections with academics	3
Assessment submission policies	7	Graduation information	3
Expectations on assessment	7	Online social networking opportunities	3
Welcome messages from Course Convenors	7	Monash terms glossary	2
Online technology requirements	6	ask.monash search engine	2
Orientation to online learning software	6	Self-care and well-being	2
How to succeed online	6	Making connections with other online students	2
General LMS support	6	Link to university employability portal	2
Technology support services	6	Information on exiting the university	2
Time management	5	Building online community guidelines	1
“Who to contact” at Monash	4	Groupwork guidelines	1
Integrity and professionalism	4	Links to indigenous units	0
Career progression information	4	Professional networking	0
		Graduate/alumni network	0

##### Outcomes

The resources appearing in Table 3 reveals many areas of duplication of content. In sum, these resources include information on assessment, as well as ‘special consideration’ procedures, academic integrity, and setting expectations, guides and instructions on using the LMS, course-related content and updates and library resources. Many courses offered introductions and welcome messages from course convenors, guidelines on how to study and succeed online, study planning, and study skills, such as writing and presentation skills. These outcomes are aligned with resources and strategies commonly reported in the literature (Carruth et al., 2010). However, the duplication of content in these areas reflects the tendency for individual courses to work in silos in developing support material for their students, and hence, potential inefficiencies and compromises to the quality of this material.

Alternatively, orientation resources surveyed but less commonly offered include content on student conduct, access to the university’s student charter, resources on professional etiquette online, online social media networking and connecting with others, and information on careers, graduation, graduate attributes, and alumni networks. A review of the perceived usefulness scores of each resource revealed that the most commonly reported resources were also perceived to be the most useful. Ultimately, the frequency and perceived usefulness of the resources surveyed depends on how relevant they were to the students’ first few weeks in their course. Resources that are valuable to students during and at the time of course completion are rarely provided.

#### Design of the Monash Online Learning Hub

##### Participants and procedure

**Fig. 3:**
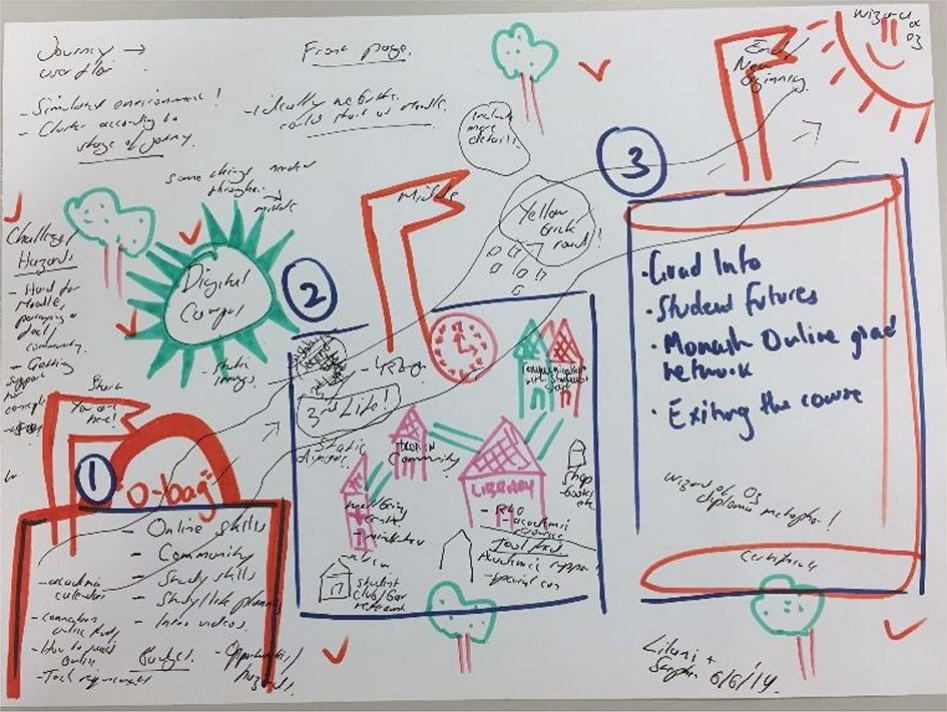
An example of a visual representation of MOLH created during the co-design workshop

To assist in the generation of appropriate, practical as well as innovative ideas around the structure of MOLH, members of the CoP were invited back to the project with an invitation to a co-design workshop, “Brainstorming Workshop II”. During the workshop, participants were initially presented with the outcomes of the baseline analysis summarised above. Participants were then asked to form groups, and use a BrainSketching technique (Van Der Lugt, 2003) to produce on paper a visual representation of how MOLH could “look”. An example of a product created during this workshop appears in the Fig. 3.

The co-design workshop served to explore ideas and thoughts around the development of MOLH, without considering constraints. For instance, the creative example in Fig. 3 contains words and metaphors such as “simulated environment” and “yellow brick road” to represented the potential functionality the site, a “you are here” navigation function, suggested imagery resembling a physical campus in digital form, and support content organised into meaningful themes that follow the usual stages of course progression. At the same time, ideas were taken from existing online orientation sites at the university, including the on-campus orientation program, called Monash Essentials. Combined, these sites would provide a broad range of options for site development, to be explored further by the Educational Designer employed to develop the site. Exploring existing sites to define the structure of programs is common practice (Cho, 2012), and provides not only a template for site development, but also enables opportunities to achieve consistency and coherence in student experiences.

##### Outcomes

The results of both the analysis and design phases of MOLH helped ascertain the main sections of content needing development. Also taken into consideration were the four pillars of student support discussed by Roddy et al. (2017), including academic support, technology support, health and well-being, and sense of community. These pillars capture the “intangibles that educators might take for granted when providing fully online courses” (Roddy et al., 2017, p. 5). As such, they too helped inform the objectives of the resource, to ensure that these intangibles are provided in a standardised fashion to all online students in the University.

Taken together, the results helped identify the following specific student outcomes that MOLH would aim to achieve for its online students. Specifically, after viewing MOLH online students are expected to:

1. Acquire knowledge of the student services available to support learning, including online learning, at the University.
2. Develop an understanding of the nature of online learning.
3. Prepare for online learning effectively.
4. Develop a sense of belonging to Monash University and its large student community.
5. Develop self-awareness and acquire effective work-study well-being practices.

#### Development of the Monash Online Learning Hub

##### Participants and procedure

The development of MOLH was initiated as soon as an Educational Designer was appointed. The Educational Designer took approximately 3–4 months at full-time capacity to develop the resource, in addition to obtaining contributions from the entire project working group and individual resource teams that were formed to create new or consolidate existing site resources. The Educational Designer’s activities, to start, included (1) a review of existing content pulled from other orientation sites of the university and a review of the results of the analysis phase, (2) a site navigation infographic of the key components of the site was created based on the outcomes of the co-design workshop with the CoP, (3) a collaborative platform was created (in Trello) to map out the skeleton of the student journey for easy dissemination, and (4) a sandpit site was created in Moodle to house potential existing and new resources during development.

The resources listed as currently offered by courses in Table 3 were organised into thematic categories by the project working group using a thematic analysis (Boyatzis, 1998). This was done manually, to identify emerging themes from the lists of resources. The aim was to structure the site around these themes, for ease of navigation for students. These themes were coded by two members of the project working group, and consensus, i.e. inter-rater agreement, was reached on 90% of the resources. For example, resources around assessment support, including study instructions, writing/presentation skills, study planning, academic integrity policies, and assessment submission guidelines, etc., were agreed as being part of the same theme of orientation content. Six themes were initially created. Two of these were merged due to significant overlaps in content (assessment resources and library study resources). The final set of five themes covered content on (1) Welcome to the University, (2) Digital literacy, (3) Study resources, (4) Well-being resources, and (5) Careers information. Resources considered to be course-specific rather than general/transferrable were not included in this thematic analysis, as they were beyond the scope of the current project.

The content development working groups were led by members of the project team, and included volunteers from the MOEC, who joined the groups based on their preferences or expertise in particular content areas. Each working group met an average of four times. During meetings, group members discussed their section of the site, reviewed any existing related content from other university orientation programs, made decisions on content selection, and where appropriate set accountabilities and action items for the generation of new content. The Educational Designer attended all meetings to ensure task focus, that the project aims were met, and that duplication or overlap in content creation was avoided.

##### Outcomes

**Fig. 4:**
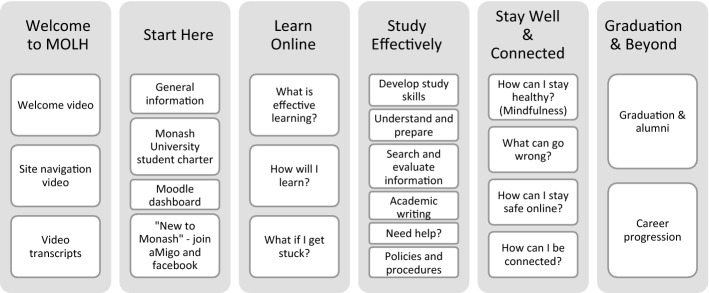
The six sections of content of the Monash Online Learning Hub (MOLH), with topics

Eight sections made up the final site, including sections representing each of the five themes, an Overview section with a site index at the start, a Start Here section, and a Questions & Feedback section at the end. Six of the eight sections contained substantial orientation content, and are presented in Fig. 4.

The six sections of content displayed above, emulate the sequence or progression of course completion, starting with general information and instructions, and finishing with graduation information and career support. In sum, each section of MOLH was informed by the results of the collaborative brainstorming and co-design workshops held with the CoP members, contained the content consolidated and created by the additional working groups dedicated to each theme, and was reviewed by the main project working group, to ensure that all requests and resources appeared as intended. Each of the sections are discussed further in Table 4.

**Table 4:** Descriptions of each of the six content sections of MOLH

Section	Name	Description
1	*Welcome to MOLH*	In this first section, students are introduced to the site, and informed of its purpose. They are initially welcomed to the site with a recorded video by a senior member of the university to support students with “institution presence” (Horvath et al., 2019), and a site navigation video to help with exploring the site fully. It is anticipated that this section of MOLH will enhance students’ connection to the wider Monash University community, contribute to students’ sense of belonging, and buffer against attrition (Oomen-Early & Murphy, 2009)
2	*Start here*	This section provides links to key information relevant for all Monash students. It includes links to *Monash Connect* (first point of contact for all student administrative enquiries), fee help, university key dates, the Student Charter, the student LMS (Moodle), and *aMigo* (new social network site for Monash students). One of the key outcomes of this section is to ensure students are familiar with University wide information and policies, ultimately increasing students’ socialisation to the university (Robinson et al., 2010)
3	*Learn online*	The three topics covered in this section are relevant to students at any stage of their online learning journey. The three topics in this section contain detailed information and videos covering; *what is online learning, how will I learn, and what if I get stuck*. This section introduces the concept of online learning and each of the main systems and programs students need to become familiar with whilst learning online at Monash. It includes a self-preparation checklist for learning online (technical), step-by-step videos demonstrating how to log into the Monash student dashboard, LMS, individual units, and various university resources to assist at any stage of their studies. Additionally, student testimonials from current Monash online students discuss what they enjoy most, what they find most challenging, and their top tip for new online students. This section is vital in an orientation site for online students (Cho, 2012; Horvath et al., 2019; Taylor et al., 2015)
4	*Study effectively*	This next section invites students to explore and further develop skills for studying, researching and writing in their online course. Links are provided to Library pages with practical tutorials on transferrable topics such as critical thinking, brainstorming and mind-mapping, referencing and more. This section also aims to help students understand their assignment tasks better, develop search strategies and use the Library databases effectively as well as the art of academic and research writing (Muping et al., 2006; Raish, & Behler, 2019)
5	*Stay well & connected*	One of the larger sections of the MOLH and primarily non-academic focused, this section explores becoming and staying physically and mentally healthy, challenges that students can face that impact on both academic and non-academic wellbeing (Johnson, 2015; Khiat, 2015), as well as being connected (Horvath et al., 2019; Roddy et al., 2017) and staying safe online. The first topic introduces students to the practice and benefits of Mindfulness and proceeds to provide a suite of tailor made mindfulness activities for students to help with study, work and life. Next, accessible descriptions of common challenges that many students experience during their studies including academic resilience, self-motivation, procrastination, perfectionism and burnout, are provided. Students can then review further information and suggestions for practical steps to help manage these challenges (Eaton et al., 2018). Lastly, this section addresses a unique challenge to online study—feelings of belonging in a student community, student support and advocacy
6	*Graduation & beyond*	The final section of MOLH celebrates graduation and student alumni. This section provides links to assist students who are nearing completion, as well as forward planners and includes information on graduation, alumni, career planning, career connect and a range of online workshops to help with career planning (Eaton et al., 2018; Horvath et al., 2019)

The MOLH features a modern illustrative and instructional design, enhanced navigation and ease of flow that incorporates special features including:

1. Progress bars within each of the six sections to indicate progression through the content,
2. A static navigation bar on the side to allow students to navigate to any of the six sections at any time,
3. A navigation video to guide learners in their search for material,
4. Access to video transcripts ensuring inclusivity,
5. Authentic testimonials from real online students sharing their experiences at the university,
6. Links to resources that open in a new browser window and do not disrupt flow, and
7. The creation or refinement of new and non-existing resources on sections and topics including: *mindfulness, online etiquette and learning online,* to name a few.

**Fig. 5:**
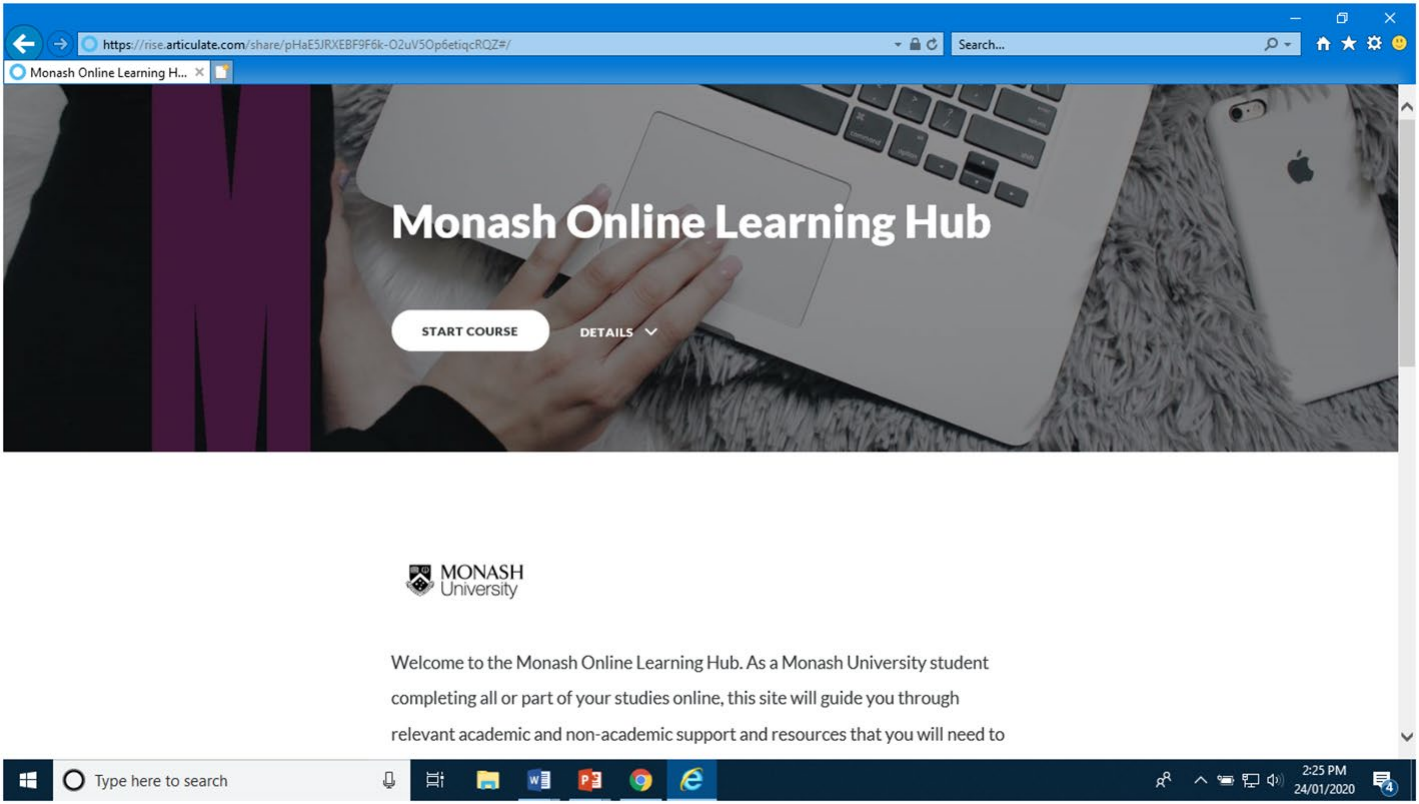
The Monash Online Learning Hub (MOLH) homepage in Articulate Rise 360

To ensure that online students received the same attention during their orientation to the University, it was considered appropriate that the MOLH should sit within the Student Engagement and Campus Experience team, of the Campus Community Division. This is the team that coordinates the university’s orientation resources for students studying on-campus, and facilitates the development and deployment of Monash Essentials—the on-campus orientation resource created for all on-campus students, domestic and international. Monash Essentials is made accessible to students several weeks prior to the start of the on-campus semesters—both Semester 1 and 2. The on-campus resources are featured within an Articular Rise 360 site, which is a web application that stores content in a way that is visually appealing and user-friendly. The site is embedded in Moodle, and is therefore accessible to all students of the University at their point of enrolment. Combining MOLH and Monash Essentials means that online students receive the same access to resources as their on-campus counterparts (Lee, 2010). It is also anticipated that by placing MOLH within Monash Essentials, online students have a meaningful and purposeful connection with the university, beyond the connections they make with their individual course of study (Pascarella & Terenzini, 2005). For these reasons, MOLH was also built in Articulate Rise 360, to emulate the experience offered by Monash Essentials, and to achieve consistency in experiences across all students. An initial draft of the site was produced and prepared for review. A screenshot of the site’s homepage is presented in Fig. 5.

#### Evaluation of the Monash Online Learning Hub

A formative review was conducted on a draft version of the site to identify areas of improvement before its deployment with Monash Essentials. Two participant groups were involved in the review—volunteer members of the CoP, and current online students.

##### Participants and procedure

Members of the CoP evaluated MOLH during a third and final workshop. As previously, members were invited to participate voluntarily. Some had participated in previous workshops related to this project, whilst others attended for the first time. Participant members were asked to review the site during the workshop, and note down their feedback in detail. They also completed a survey with 22 closed questions on a five-point Likert scale, ranging from 1—strongly disagree to 5—strongly agree. The survey was adapted from Kay (2011) and Marton (2000), and aimed to obtain quantitative feedback about the useability, accessibility, content and learning, and engagement of the MOLH. The questions and results of the survey are displayed in Table 5. After completing the survey alone, participants had an open discussion, during which they shared their ideas and feedback, and responded to the feedback of others.

**Table 5:** Aggregate results from MOEC members and online student participants on the 22-item MOLH evaluation survey

Question (this site was:)	Staff (*n* = 7)		Students (*n* = 5)	
	Mean	SD	Mean	SD
Usability				
Easy to use	5.0	0.0	4.4	0.5
Simple to understand	4.9	0.4	4.6	0.5
Quick to complete	4.6	0.5	3.0	1.6
Logically structured	4.6	0.5	4.4	0.5
Accessibility				
Entry points to visit this site were easily accessible	4.9	0.4	4.2	0.8
Locating information was easy	4.6	0.5	4.0	0.7
Navigating across the content was smooth	4.4	1.1	3.6	1.5
Going back to previous sections was easy	4.9	0.4	4.2	0.4
Content				
Content addresses broad range of relevant topics	5.0	0.0	4.6	0.5
The content was detailed enough	4.4	0.8	4.0	1.2
The site provided useful information	4.7	0.5	4.2	0.8
The site content presented information & resources I had not considered before	3.6	1.5	4.0	0.7
The content was well organised	4.9	0.4	4.6	0.5
The was some inconsistency in the site	2.7	1.5	2.4	1.0
Learning and engagement				
The information and resources in the site helped me learn	4.4	0.8	3.8	1.5
The site was engaging	4.4	0.8	4.4	0.9
I would like to return to this site during my studies	4.3	1.0	4.4	0.9
I would recommend this site to others	4.6	0.8	4.4	0.9
I did not learn something new	2.0	0.8	1.6	0.5
I lost interest pretty quickly	1.6	0.8	2.2	1.3

MOLH was also shared with a group of current Monash University online student participants. Student participants were recruited through convenience sampling, via their course leaders. Of a total of 19 students who initially expressed interest in taking part in the study, only five attended the interviews, all of whom were female. At the conclusion of the interview, participants were thanked and each offered AUD$50 Coles gift cards.

The semi-structured interviews were guided by an interview schedule that utilised discussion strategies and open-ended questions as set out in Bhattacharya (2017), to enable the exploration of further ideas and insights using probing questions, such as “why” and “why not” (Tong et al., 2007). Specifically, the interview schedule consisted of nine open-ended questions capturing three themes of content, the results of which appear below. Participants were then allowed 15 min to go through MOLH alone, before responding to the same 22-question survey completed by members of the CoP.

##### Outcomes

The majority of CoP members fed back how useful they found MOHL, describing it as a “one stop shop” for much needed information for online students. Key suggestions of improvement were made on incorporating Indigenous population information to enhance inclusiveness towards students including minorities, to include items for international students, and to emphasise the benefits of studying online. Another recommendation from CoP staff was to integrate a search tool function that is easily discoverable or a map of MOHL and its webpages to assist with easier navigation of the webpage.

Students, on the other hand, provided the following feedback on how well the site met their needs, the overall strengths and weaknesses, and suggestions for improvement:

- *Reflecting on academic needs*
  To start, participants reflected on their general academic needs as online students, and in doing so referred to the need for having engaging teaching staff (n = 3). Course leaders were expected to be willing and available to provide prompt responses with academic advice (n = 2), as well as guidance on how to perform well in their course of study (n = 1). A participant listed “excellent feedback, approachability, prompt replies via email, and willingness to do a Zoom call on a daily basis” as characteristics they would like to see from their course instructor. The recurring non-academic needs of students that arose comprised access to technical support such as how to navigate Moodle and deal with IT issues (n = 4). In relation to this, one participant stated the availability of technical support to be “a significant gap, especially for new students” as online students usually study outside the hours that technical support is provided. Other notable needs included a preference for interaction with other students, whether that be on Facebook or organising a group meetup in person (n = 3) some stated a need for “interaction with other students”, such as through study groups. Finally, some students mentioned the need for mental health support during study (n = 2). Overall, the results echo the findings found in the literature which capture the prominent needs of online students (listed in Table 1).
- *Perceptions of the strengths and weaknesses of MOLH*
  The initial response of participants to MOHL was very positive. All participants (n = 5) noted the breadth of information available in a single location as a key highlight of the site. One participant’s response was that “..it’s very clear, well set out, you can navigate through it easily”. MOHL was perceived differently to other online learning platforms that students used such as Moodle. MOHL was more logical, clear, comprehensive, collated, and streamlined than Moodle (n = 4). MOHL allowed participants to search for much sought information without having to reach out to their course specific study advisors. Some participants indicated that the site was easy to use (n = 3), whereas others indicated that there was a lot of scrolling to get to the information required and could maybe be better mapped (n = 3). A participant stating “Probably the only criticism I’ve got is there’s too many click throughs, you click too many times to get to the content. I think it’d be easier if you click once and all the content just comes up.” MOHL overall was perceived as user friendly as the main landing page is inviting and insightful (n = 2), with the side navigation panel being noted helpful in directing through the website (n = 2).
- *Suggestions for improvement*
  Participants expressed being happy with the site as it is, and some did not have any suggestions for improvement (n = 2). Some did suggest that MOHL and its contents could be further advanced by adding more library resources such as writing tips, sample papers/assessment pieces, academic exercises, workshops, and tools to assist with critical thinking (n = 2). An example is one participant voiced “I’d like to see more of the library resources...adding some of those things in there would be really useful”. They also voiced a request for a more prominent search toolbar (n = 2).
  The results in the table above reveal that students and staff rated the MOLH positively with regard to its usability. They largely agreed that the content was relevant, detailed, useful, and well organised. Encouragingly, participants agreed that the MOLH resource would be something they would return to and recommend to peers. It should be noted, however, that student and staff participants provided the above feedback without knowing the exact capabilities and restrictions of Articulate Rise as a platform, and without knowledge of the context within which the MOLH was to be embedded, i.e. as a standalone site or alongside other course-specific orientation resources. A pilot study of the MOLH after implementation is needed to obtain more specific and appropriate feedback of its distinct value.

## Lessons learned, recommendations and conclusions

### Lessons learned and recommendations

The collaborative approach adopted in this project made an overall positive contribution to the final product. There were, however, times when not all ideas were achievable and not all preferences were fulfilled. For instance, it was noticed that attempting to obtain creative and innovative ideas from the community of practice in the brainstorming and co-design workshops led to unmet expectations by the same participants who reviewed the site in a subsequent workshop. Instead, innovative ideas around the look, feel, and impact of the site were compromised for reasons relating to practicality and feasibility. For example, it was suggested in the first brainstorming workshop with the CoP, that the use of AI and VR features would enhance the personalisation of the student experience and immersion with the university. Whilst the inclusion of these features would have been ideal, more important was feasibility within our timeframe to explore these ideas or not, and the cohesiveness and complementarity of the resource with the on-campus equivalent. As such, we recommend that the use of utopian activities to arouse divergent thinking and creativity in co-design workshops be tempered with the setting of realistic expectations of what can be achieved.

Another recommendation would be to consult online students during the *needs analysis* phase of the project (as done by Horvath et al., 2019). In the current project, a needs analysis was conducted with staff only, albeit experts in online education and members of the community of practice, to provide insight into the teaching and learning practices and resources needed to support online education students. It was also deemed sufficient at the time to consult existing literature on online student needs, as done by Cho (2012), as this literature is available and detailed enough. Although the analysis provided a range of categories and resources coupled with results from a literature review, due to time constraints, the needs of Monash University students in particular, voiced by students themselves, were not included until the final stage of site development. It could be that the needs of online students at Monash University, by virtue of the demographics of these students, and perhaps the location and culture of the university, different needs might have arisen from those familiar to staff and reported in the literature. An assumption was made that all online students have similar needs, which may be worth challenging. Irrespective of whether findings from students at Monash University do or do not differ from those in the literature, we would recommend that the student voice be heard during a needs analysis of future orientation site design phases.

Whilst a 30% response of online education staff participating in the baseline analysis to gauge current practice was acceptable, it reflects potential areas of improvement in processes. Specifically, a clearer definition (inclusion criteria) for what orientation material actually is would have been helpful. It was noted during the baseline analysis that there was some confusion as to what constitutes an orientation resource, and what was it that we were looking for in the attempt to gather and consolidate existing resources. Ultimately, leaders of the five working groups developing content for MOLH ended up accessing a great variety of orientation sites from participants to make the decision of what was and what was not appropriate, instead of these being provided to them by course teams, which would have been more efficient. The exercise did, however, show that the university offers an abundance of resources to students, and putting them all together in a cohesive way is not a simple task given that huge variety.

### Conclusions and future directions

University programs of study that adopt an online mode of delivery, either fully or in part, are becoming increasingly more popular, and open the door to a wider and more versatile use of space and time for teaching and learning to take place. While universities have traditionally invested considerable time and resources to the orientation of students studying online, there is growing recognition of the need to provide comparable experiences to students studying fully or partly online.

This collaborative project was developed by a core team from the CoP, an established community of practice at Monash University that was devoted to online learning. In fact, the creation of MOLH would not have been possible without extensive inter-faculty collaboration. Substantial time was taken to engage with members of the CoP outside the core project team. Having a dedicated and diverse group of academics invested in online education was critical to the ideas generation, resource creation, and review phase of the project, and has been incredibly invaluable. This has not only strengthened the final product and ensured it has wide relevance across the university but also served to build engagement through the community of practice. That is, this tangible example highlights that activities of the CoP have gone beyond providing a forum for information sharing and networking, to creating value that directly enhances student learning and well-being, and organizational outcomes (Wenger et al., 2000).

The main conclusion we can draw from this case study, therefore, is the value of applying such a collaborative and rigorous framework to create a resource that is fit for purpose. Both academics and students found the site relevant, detailed, useful, and well organised. As outlined above, each phase of this project was clearly structured and designed to harness the collective expertise of an online community of practice, draw together existing yet disparate resources across the university, and collaborate in creating a cross discipline resource relevant for all online students. The project resulted in the resource being successfully integrated into the University’s mainstream student orientation platform. This case study and its achievements substantiate the approaches taken in previous work (e.g., Cho, 2012; Horvath et al., 2019; Taylor et al., 2015).

In particular, the resource will undergo a pilot test in 2020, i.e. a summative evaluation, in an effort to systematically establish the value and the anticipated student outcomes of the module. Indeed, there is much to be learned from the roll out of MOHL. At a very basic level, it will be valuable to understand rates of uptake/access of the resource, by students of different backgrounds and in different disciplines. Beyond that initial access, learning analytics has the potential to provide valuable insight into how students engage with the material, what attracts their attention when first accessing the site and what resources do they return to over the course of their studies depending on their needs (e.g. accessing resources on workload management or wellbeing at a time then they are faced with multiple demands on the home, work and study fronts).

The assessment of student engagement with the resource in the short term needs to be complemented by longer term evaluation to understand the impact of orientation resources like this on student retention and the quality of their overall learning experience (Horvath et al., 2019). Given the rising importance of online learning and teaching, there is much to be learned from the rigorous development and evaluation of initiatives such as this since the insight has the potential to enhance practices and student learning around the world.

The funding provided to create the Monash Online Learning Hub covered the development of a single version of the resource. However, the resource will require revision and re-development in the near future, as content becomes outdated, and as new ideas to improve the site emerge. Furthermore, the resource will evolve as online resources become applicable to all modes of study, beyond just the online mode. For example, even if a course is taught in an on-campus mode, there will likely be some online component e.g., an LMS which houses resources. So while there has previously been a strict gap between on-campus and online modes, this may increasingly dissolve in some areas. In other words, what is relevant for an online student may well be relevant for those on-campus. This was a consideration in our case, as we needed to integrate in some capacity the MOHL with the on-campus Monash Essentials orientation site, to instil inclusivity and belonging of online students to the wider student community (Lee, 2010; Pascarella & Terenzini, 2005). Such an integration will further reduce the risk of duplication in areas of overlap between on-campus and online. Another example of this transferability, which has just recently emerged, concerns the current bridge that the online resources have provided to support students affected by COVID-19 travel bans. With students unable to travel to campus, MOHL is being used at this critical time, when resources need to be adapted for online use, and on-campus processes need to be converted into online resources, offering support.

Finally, the MOLH will be launched officially in mid 2020, with links to the final version to be included in Monash Essentials, the university-wide on-campus orientation program. Online students will receive access to Monash Essentials upon enrolment, and direct links to MOLH will be distributed across the great variety of courses with online components that exist at the university. As suggested by others (Herodotou et al., 2020; Horvath et al., 2019), the launch and wider implementation efforts of the MOLH will reinforce the institution-wide commitment to meeting the needs of all students regardless of study mode, and policies and procedures around quality orientation and transition should be a priority online just as much as it is on-campus.
